# Pharmacokinetics of calycopterin and xanthmicrol, two polymethoxylated hydroxyflavones with anti-angiogenic activities from *Dracocephalum kotschyi Bioss*

**DOI:** 10.1186/s40199-016-0161-x

**Published:** 2016-10-04

**Authors:** Seyedeh-Somayeh Zamani, Mohsen Hossieni, Mahmoud Etebari, Pirooz Salehian, Soltan Ahmad Ebrahimi

**Affiliations:** 1 Department of Pharmacology and Toxicology, Isfahan Pharmaceutical Sciences Research Center, School of Pharmacy and Pharmaceutical Sciences, Isfahan University of Medical Sciences, Isfahan, Iran; 2 Department of Pharmaology, School of Medicine, Tehran University for Medical Sciences, Tehran, Iran; 3 Sarem Fertility and Infertility Research Centre, Sarem Hospital, P.O. Box 1396956111, Shahrak-e-Ekbatan, Tehran, Iran; 4 Department of Pharmacology, Iran University for Medical Sciences, Tehran, Iran

**Keywords:** Calycopterin, Xanthomicrol, Flavonoids, *Dracocephalum kotschyi*, Pharmacokinetics

## Abstract

**Background:**

Recently flavonoids have attracted the attention of researchers in the fight against cancer. Calycopterin and xanthomicrol, are two polymethoxylated flavonoids found in the aerial parts of *Dracocephalum kotschyi* Bioss.. We have recently shown that these compounds possess antiangiogenic activity and may be of value as potential anticancer agents. In order to demonstrate putative in vivo antitumor effect of these compounds we needed preliminary information on both pharmacokinetics and toxicological properties of these two agents.

**Method:**

A new online SPE HPLC method for measurement of calycopterin and xanthomicrol in rat plasma was developed. Pharmacokinetic parameters of calycopterin and xanthomicrol, after i.v. administration in rats, were determined.

**Results:**

The plasma half-life for both agents was around 4 h, however, the volume of distribution of calycopterin appeared to be about 8 times greater than xanthomicrol. This was probably due the greater hydrophobicity of the former which had other consequences such as much smaller maximum plasma concentration of calycopterin compared to its less methoxylated congener. Preliminary toxicological study of xanthomicrol failed to show any behavioral, histological and biochemical adverse effects after repeated administrations of high doses.

**Graphical Abstract:**

Pharmacokinetics of xanthomicrol in rats.
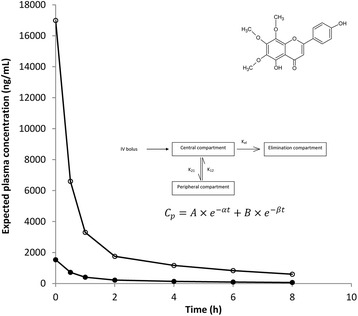

## Background

**Fig. 1:**
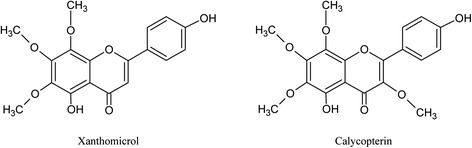
Structures of calycopterin and xanthomicrol

The demonstration of antiproliferative effects of a number of flavonoids against cancer cell lines, has attracted the interest of researchers in these chemicals as potential therapeutic agents for the prevention and/or treatment of different forms of neoplasms [1]. The in vitro effects of flavonoids range from being generally cytotoxic [2] to showing selective effects against some cell lines and not others [3]. The molecular mechanisms involved in the cellular effects of flavonoids reflect a similar diversity: from a change in inner mitochondrial membrane permeability leading to cell death by many flavonoids [4] to selective regulation of the miR-101/MKP-1/MAPK pathway to decrease the inflammatory response [5] by genkwanin. Structure activity relationship (SAR) studies have been able to delineate the link between molecular structure and selectibity of cytotoxic effects for some of these compounds [6]. For example, data obtained by Moghadam et al. [6] suggests that polymethoxylated hydroxyflavones like xanthomicrol and calycopterin (Fig. 1) appear to have more selective activities against cancer cell lines compared to hydroxyflavones with no methoxy groups such as luteolin or apigenin. It can be reasoned that a xenobiotic with across the board cytoxicity does not get us any nearer to finding new therapeutic agents with less side-effects than the currently used drugs and thus the more selective agents should be better candidates for further research. However, as our knowledge about the in vitro mode of action of these agents increases, we obtain a better understanding why those flavonoids which are not globally cytotoxic against a multitude of malignant cell lines, may help decrease tumor size in vivo and therefore have potential anticancer activity with little or no direct cytotoxic effects on neoplastic cells making up the bulk of the tumor. Recent work in our laboratory on calycopterin and xanthomicrol has shown these compounds to possess, in addition to their selective cytotoxic effects, potent antiangiogenic activities which appear to be due to inhibition of endothelial cell proliferation via decreased VEGF activity [7]. The importance of angiogenesis in tumor growth and metastasis was first suggested by Folkman [8] and later demonstrated by many other workers [9–11] and may provide an explanation for the antineoplastic effect of many flavanoids e.g., genistein [12]. It is also worth remembering that currently there are a number of agents under clinical trial as antitumor drugs with this mechanism of action [13].

The demonstration of in vitro antiangiogenic activities for these two polymethoxylated flavones has to be followed by investigations into the in vivo antitumor effects of xanthomicrol and calycopterin. However, these efforts are hampered by a lack of pharmacokinetic and toxicological data about these agents. In other words, we not only have to obtain data which links administered dose to plasma flavonoid concentrations but also have to provide preliminary evidence on the lack of overt toxicity. This study was undertaken to achieve these goals.

## Methods

### Plant material

Aerial parts of *Dracocephalum kotschyi* Bioss. were obtained from a herbalist in the city of Isfahan and the identity of the specimen was confirmed by Dr Gh Amin, Faculty of Pharmacy, Tehran University of Medical Sciences (voucher specimen number at the faculty of pharmacy herbarium: PMP-304).

### Isolation and purification of xanthomicrol and calycopterin, identity confirmation, recovery calculations

Xanthomicrol and calycopterin were isolated and purified as previously reported [6] with some modifications to the HPLC step. Briefly, 10 kg of dried aerial parts of Dracocephalum kotschyi Bioss. were manually powdered and extracted in 50 g batches by overnight extraction with 400 mL ethyl acetate using a Soxhelt apparatus. The extract aliquots from 10 kg plant material were dried in vacuo, combined and finally dissolved in 8 L chloroform and filtered through Whatman No. 1 filter paper to remove particulate matter. The flavonoid content was extracted in 1 L batches into 1 L 1 M ammonia solution. The intensely yellow ammonia solution was acidified to pH 1 using concentrated HCl and the flavonoid content was back extracted into 200 mL ethylacetate. The non-methoxylated hydroxyflavones were removed by treatment with alumina. The organic solvent was evaporated in vacuo. The material obtained in this step was subjected to semipreparative HPLC purification as follows. A 10 μm Nucleosil ODS column from Macherey Nagel (21x250mm) was equilibrated with a mobile phase containing 37 % ACN, 24 % MeOH, 38.5 % Water and 0.5 % triethylamine, adjusted to pH 6 with acetic acid. A saturated solution of the extract in mobile phase was centrifuged at 10000 g for 5 min and 1.7 mL of the supernatant was injected onto the column. The column eluent (5 mL/min) was monitored at 226 nm using a LKB Uvicord UV detector connected to a Younglin (Younglin, South Korea) data acquisition module. Fractions containing xanthomicrol, calycopterin and circimaritin were collected and dried by lyophilization. Peak identities were confirmed by infrared spectroscopic analysis [6]. The flavones content (μg per gram of dried aerial parts of the plant material) was calculated. Purity of isolated compounds was examined using analytical HPLC on a 4.6x150mm, ODS-2, 5 μm, Tracer Excell column (Technochroma, Spain) using the same mobile phase as described above, pumped isocratically while detecting at 263 nm.

### Analytical HPLC method for measurement of xanthomicol and calycopterin

Confirmation of calycopterin, xanthomicrol and circimaritin identity was carried out using HPLC analysis of purified compound compared to the material obtained from previous work [6] and also IR spectroscopy. A HPLC method with online solid phase extraction was developed for the analysis of xanthomicrol and calycopterin in biological fluids (Fig. 1) using circimaritin as internal standard (IS). 100 μL of plasma and 10 μL of 500 μg/mL IS were mixed in a 2 mL microcentrifuge tube. 40 μL of 10 mg/mL ZnSO_4_ was added to the tube and the content was mixed. After addition of 200 μL acetonitrile, the tube was placed in an orbital shaker for 10 min. The samples were subsequently centrifuged at 14000 g for 10 min the supernatant was subjected to online extraction/HPLC analysis as follows:

At the start of every run, the sample injection valve and the sample extraction valve were switched to load position (Fig. 1). 250 μL of sample was injected into the sample loop of the sample injection valve. The valve was switched to the inject position which diverted the flow from the online SPE (solid phase extraction) solvent delivery pump (500 μL/min) through the sample loop which in turned carried the sample onto the online SPE column (3×15mm, ODS-2, 5 μm). After 2 min, the sample extraction valve was switched to inject position. This allowed the mobile phase from the analysis solvent delivery pump (1 mL/min) to wash any material retained by the online SPE system, onto the analytical column (4.6×150mm, ODS-2, 5 μm, Tracer Excell, Technochroma, Spain) for separation and measurement. The mobile phase pumped by online SPE solvent delivery pump consisted of acetonitrile:water (35:65) and the mobile phase pumped by the analysis solvent delivery pump was composed of acetonitrile:methanol:water:triethylamine (37:24:38.5:0.5). 16 min after the start of analysis, the mobile phase pumped by the analysis solvent delivery pump was switched to methanol:tetrahydrofurane:dichloromethane (80:10:10) for 3 min. This allowed for highly non-polar compounds retained by the SPE and analytical column to be flushed. Detection was at 263 nm.

The assay system was calibrated for both xanthomicrol and calycopterin over the concentration range of 125 to 1500 ng/mL. In order to estimate the recovery of the assay, the calibration curve with online SPE was compared to a standard curve injected directly into the analytical column, without the SPE step. Inter-day and intra-day variation and S/N ratio were calculated for this analytical system.

### Rat femoral vein cannulation

All procedures involving animals were carried out after the approval and under the supervision of the Ethics Committee for Animal Expreiments of Iran University for Medical Sciences. In order to be able to withdraw blood samples at predefined time points during pharmacokinetic studies, femoral vein of male Wistar rats, in the weight range of 280 to 300 g, were catheterized, under anaesthesia, using the method described by Jespersen et al. [14]. After the placement of the catheter, the animals were allowed to recover for 24 h, during which time, the rats were checked for signs of bleeding and the catheters were checked for patency.

### Pharmacokinetic study

Xanthomicrol or calycopterin was dissolved in 200 μL of dimethylsulfoxide (DMSO) and injected into the dorsal tail vein of rats. Just before drug injection (time zero) and at 1, 5, 10, 20, 60, 120 and 240 min after drug injection, 300 μL of blood were withdrawn via the femoral vein which had been catheterized previously. In order to prevent phlebotomy induced hypovolemia, 200 μL normal saline was injected via the femoral catheter after each blood sample withdrawal. Blood samples were centrifuged at 4 °C for 10 min at 4000 g. Plasma was collected and stored frozen at -80 °C until needed.

### Toxicological studies in mice

Toxicity studies were carried out using Balb/C mice in the 25-30 g weight range. Drug administration was via the I.P. methoud. Animals were assigned to five groups of three: one-Control groups which received no treatment, two-Vehicle control which received 60 μL of DMSO (dimethyl sulfoxide) for 6 days, 3- Test group one which received 30 mg/kg body weight xanthomicrol in 60 μL of DMSO per day, 4- Test group two which received 40 mg/kg body weight xanthomicrol on 60 μL of DMSO per day and 5- Test group three which received 50 mg/kg body weight xanthomicrol on 60 μL of DMSO per day. The animals were weighed and examined for changes in skin tone, signs of fur loss and changes in bowl function [15]. On the seventh day, the animals were anaesthetized under an atmosphere of chloroform. Once fully anaesthetized, blood samples were collected from the heart and the animals were killed by cervical dislocation. Serum samples were analyzed for hepatic enzymes AST and ALT [16] and also for creatinin levels [17]. Kidneys, liver, lung, intestines and stomach were removed from animals and placed in 10 % formalin solution for subsequent paraffin embedding, sectioning, staining and histological studies. Tissue sections were examined for fibrotic, inflammatory and vascular changes by a qualified pathologist [18].

## Results

### Isolation and purification of xanthomicrol, calycopterin and circimaritin, identity confirmation

**Fig. 2:**
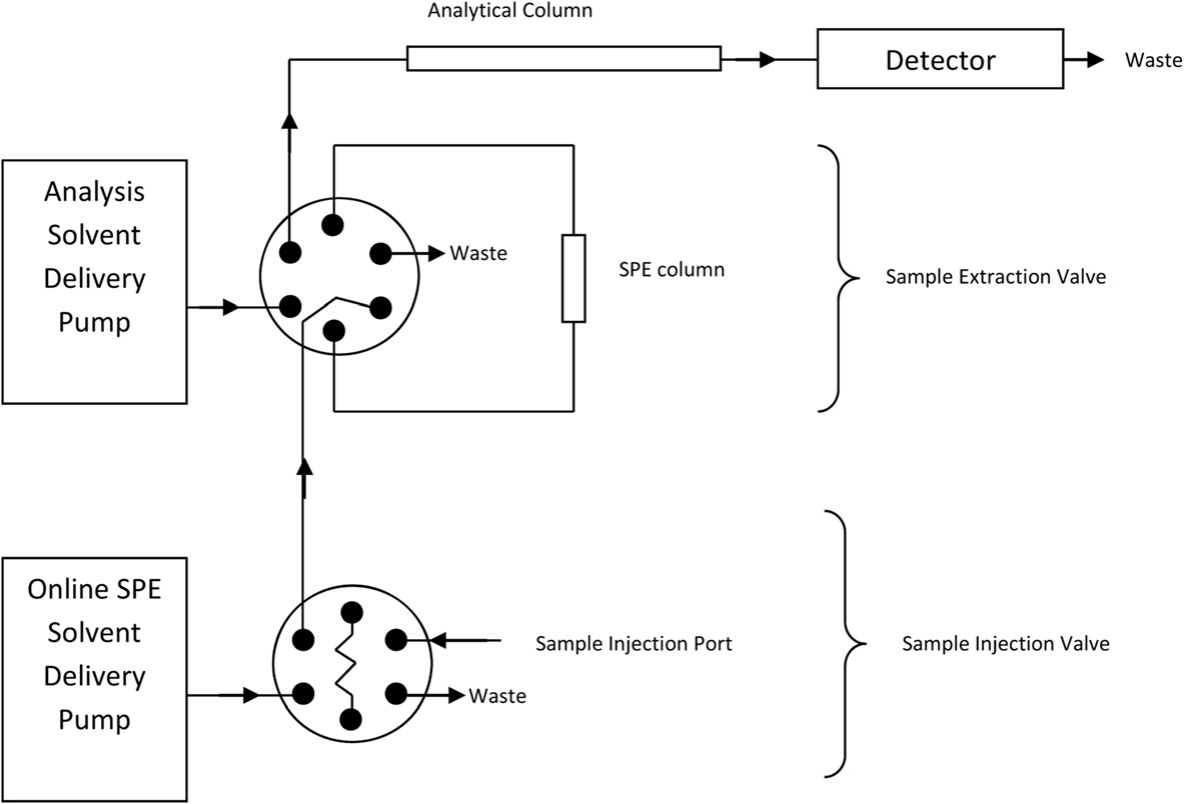
Schematic diagram of the fluidics of the online solid phase extraction HPLC system

Processing of 10 kg of dried aerial parts of *Dracocephalum kotschyi Bioss*. yielded 476 mg of xanthomicrol, 190 mg of calycopterin and 60 mg of circimaritin. The compounds obtained appeared to be pure as demonstrated by the existence of single peak in HPLC analysis (Fig. 2). The identities of isolated flavones were confirmed HPLC analysis and IR spectroscopy in comparison with standard material previously obtained [6]. The flavones contents were determined (Table 1).

**Table 1:** Flavone content of *Dracocephalum kotschyi Bioss*. dried aerial parts

Flavone	Specific amount (μg flavone / g plant material)
Xanthomicrol	95.2
Calycopterin	38
Circimaritin	15

### Analytical HPLC method for measurement of xanthomicol and calycopterin

**Fig. 3:**
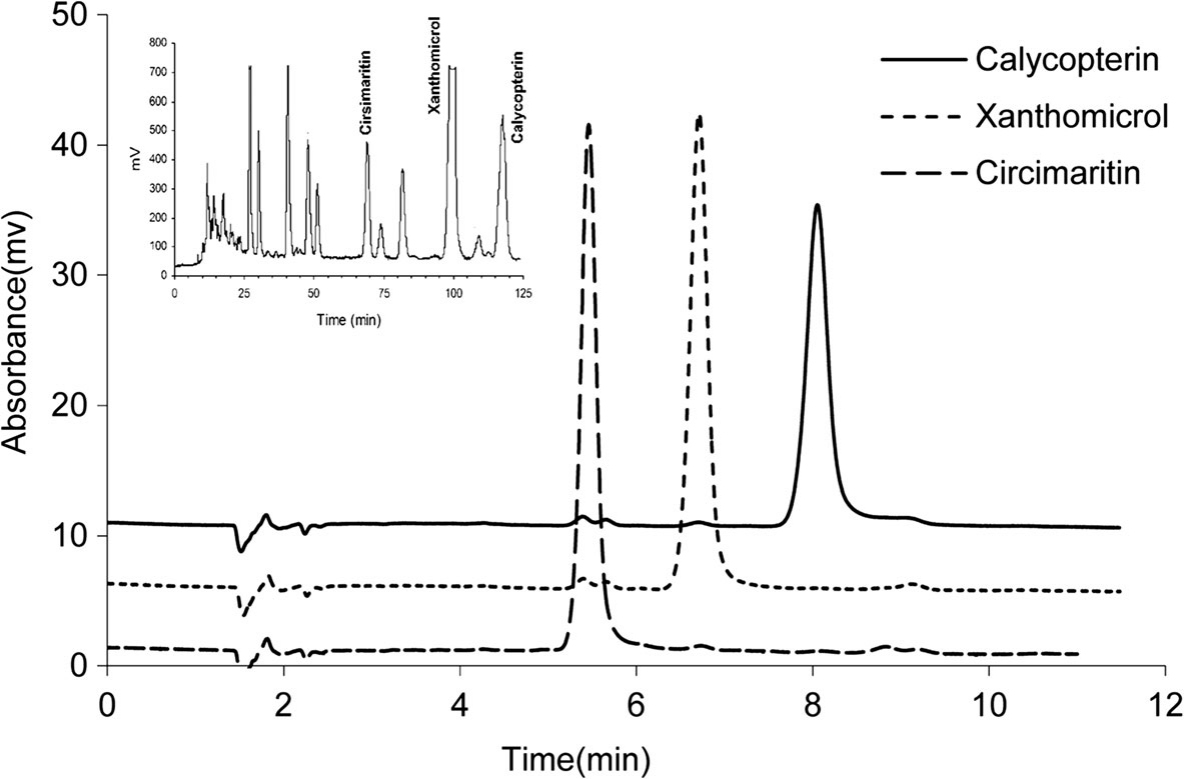
Purity of isolated flavonoids as determined by analytical HPLC. The inset shows a preparative chromatogram of *Dracocephalum kotschyi Bioss*y flavonoid fraction

Plasma samples spiked with calycopterin (125 to 1500 ng/mL) or xanthomicrol (125 to 2000 ng/mL) were extracted as described above and subsequently analyzed by analytical HPLC using online extraction (Fig. 3). Both calibration curves were linear over the concentration ranges described (Table 2). Recovery of the extraction method was investigated by comparing the peak areas of 500 ng/mL of calycopterin and xanthomicrol and 1430 ng/mL circimaritin (as IS) obtained from injection of mixtures using the on-line SPE method with the same flavone mixtures injected directly onto the analytical column, without the online solid phase extraction step. Recoveries of calycopterin and xanthomicrol were 94 % and 92 % respectively.

**Table 2:** Validation parameters for the HPLC assay of calycopterin and xanthomicrol

Compound	Calibration Equation Y = mX + C, R^2^	Interday at 500 ng/mL	Intraday at 500 ng/mL	LOD (ng/mL) (S/N) = 3	LOQ (ng/mL) (S/N) = 10
Calycopterin	Y = 0.0002X + 0.0014	4.3 %	2.5 %	10	34
	R^2^ = 0.998				
Xanthomircol	Y = 0.0001X + 0.0023	3.9 %	2.2 %	8	32
	R^2^ = 0.999				

S/N, signal to noise ratio

### Pharmacokinetic study

**Fig. 4:**
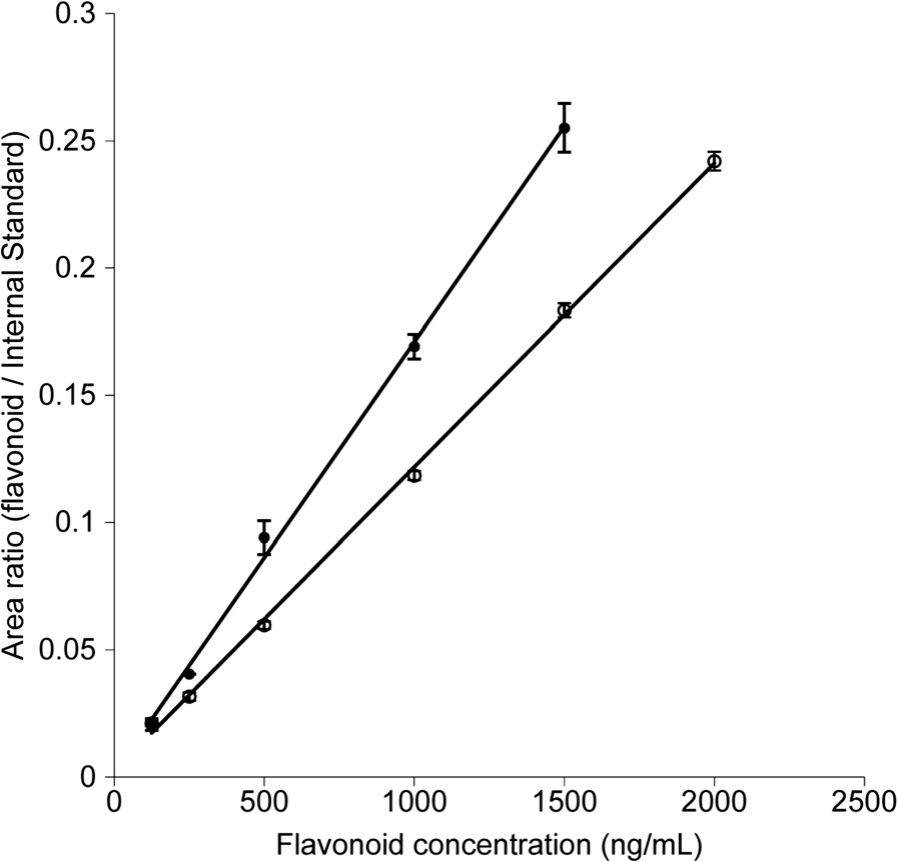
Calibration curve for xanthomicrol (○) and calycopterin (●) spiked in plasma. The values are mean ± SEM for three independent analyses

**Fig. 5:**
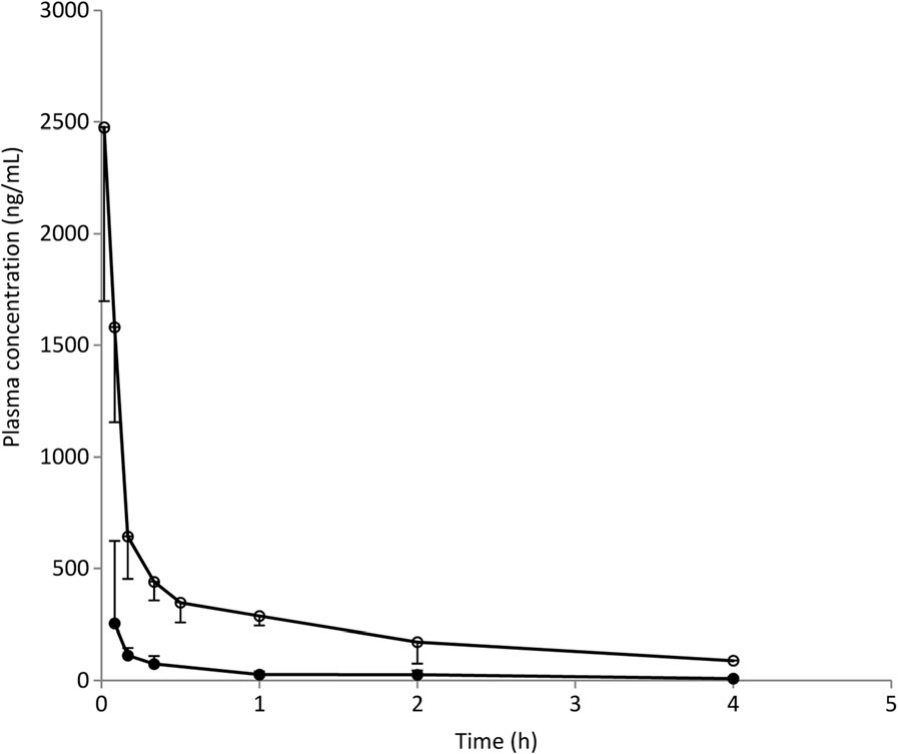
Plasma concentration-time profile of calycopterin (●) and xanthomicrol (○) in rats after IV administration of 20 mg/kg of the flavnoid. Values are mean ± SEM, *n* = 4

Figure 4 shows the plots of plasma flavones concentration against time for both xanthomicrol and calycopterin. For both xanthomicrol and calycopterin, two-compartment models were found to adequately describe the changes in concentration with time (Fig. 5). Pharmacokinetic parameters obtained from constructing the residual concentration verses time curve for these two-compartmental models are presented in Table 3. Rate constants for transfer between the central and peripheral compartments (K_21_and K_12_) and also the elimination process (K_el_) were also calculated.

**Table 3:** Pharmacokinetic parameters for xanthomicrol and calycopterin after i.v. bolus administration to rats

	C_0_ (μg/L)	V_c_ (L)	t_1/2_ (h)	K_21_ (h^-1^)	K_12_ (h^-1^)	K_el_ (h^-1^)	Cl_a_ (L/h)
Calycopterin (20 mg/kg)	312	63.9	3.8	0.52	1.39	0.26	16.8
Xanthomicrol (20 mg/kg)	2345	8.5	4.2	0.46	1.88	0.21	1.8

C_0_: Initial plasma concentration; V_c_: Volume of distribution of the central compartment; t_half_: Biological half-life; K_21_: Rate constant of transfer from the central compartment into the peripheral compartment; K_12_: Rate constant of transfer from the peripheral compartment into the central compartment; K_el_: Rate constant of elimination from the central compartment, Cl_a_: Clearance rate of the central compartment

### Toxicological studies in mice

There were no significant differences in serum creatinin content, alanine transaminase (ALT) and aspartic acid transaminase (AST) activities between control, vehicle and test groups of mice which had received xanthomicrol at 30, 40 or 50 mg/kg body weight (Table 4). The weight of animals in control group (27.1 g ± 1.2), on different days, were not significantly different compared to the animals in xanthomicrol groups (27.7 ± 1.7 g), on corresponding days.

**Table 4:** Effects of different doses of xanthomicrol on kidney and liver functions

Group	Serum Creatinin (mg/mL)	Serum ALT (IU/L)	Serum AST (IU/L)
Control	0.36 ± 0.019	43 ± 15	180 ± 48
Vehicle	0.35 ± 0.015	49 ± 29	228 ± 94
30 mg/kg Xanthomicrol	0.36 ± 045	38 ± 8.8	138 ± 37
40 mg/kg Xanthomicrol	0.35 ± 0.036	22 ± 19	130 ± 49
50 mg/ kg Xanthomicrol	0.39 ± 0.02	30 ± 3.0	316 ± 163

**Fig. 6:**
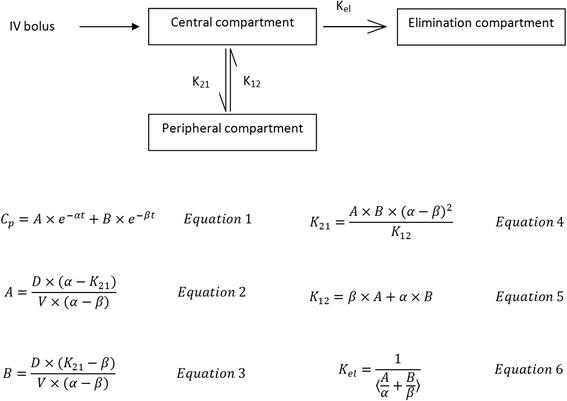
Schematic representation of the two-compartment pharmacokinetic model used to analyse the concentration data. C_p_: Plasma concentration; V: Volume of distribution of the central compartment; D” Drug dose, K_21_: Rate constant of transfer from the central compartment into the peripheral compartment; K_12_: Rate constant of transfer from the peripheral compartment into the central compartment; K_el_: Rate constant of elimination from the central compartment. A, B, α,β are hybrid constants. A and B are estimated from the intercepts on log plasma concentration axis of the best fit line of the distribution phase and elimination phase respectively. α and β are estimated from the slopes on log plasma concentration axis of the best fit line of the distribution phase and elimination phase respectively

Histological examination of tissue samples from kidney, small and large intestines, lung and heart of test animals showed absence of significant pathological change in animals receiving xanthomicrol compared to the control group (Fig. 6) (Table 5).

**Table 5:** Effects of different doses of xanthimcrol on tissue histology

Animal	Tissue	Inflammation structural change	Fibrosin	Vascular change	Notes
Vehicle 1	Lung/Liver/Heart/ Kidney	Absent	Absent	None	Normal
Vehicle 2	Small intestine/ Large intestine/ Gastric wall	Focal inflammation in gastric wall	Absent	None	Normal
Vehicle 3	Lung/Liver/Heart/ Kidney	Absent	Absent	Hypermic	Increased type II pneumocytes
Test group 1,1	Small intestine/ Large intestine/ Gastric wall	Absent	Absent	None	Normal
Test group 1, 2	Lung/Liver/Heart/ Kidney	Absent	Absent	None	Normal
Test group 1, 3	Small intestine/ Large intestine/ Gastric wall	Absent	Absent	None	Normal
Test group 2, 1	Lung/Liver/Heart/ Kidney	Focal inflammation in lung tissue	Absent	Increased vascularization in lung sample	Increased type II pneumocytes
Test group 2, 2	Small intestine/ Large intestine/ Gastric wall	Absent	Absent	None	Normal
Test group 2,3	Lung/Liver/Heart/ Kidney	Lymphocyte infilteration in lung sample, Subcapsular inflammation in liver sample	Absent	Hypermic	Normal
Test group 3,1	Small intestine/ Large intestine/ Gastric wall	Absent	Absent	None	Increased type II pneumocytes
Test group 3,2	Lung/Liver/Heart/ Kidney	Focal inflammation in lung sample	Absent	None	Increased type II pneumocytes
Test group 3,3	Lung/Liver/Heart/ Kidney	Absent	Absent	None	Normal

## Discussion

The research into the proposed antineoplastic properties of Spinal-Z, an Iranian herbal remedy, composed of peganum harmala seeds extracted and *Dracocephalum kotschyi Bioss*. leaves extract has been going on in our laboratory for more than 15 years. The inhibitory effects of β-carbolines found in peganum harmala seeds on topoisomerase activity [19] was demonstrated first, however, further work suggested a more prominent role for selective cytotoxic activity of flavonoids found in *Dracocephalum kotschyi Bioss*. leaves extract [20]. Recently we were able to show calycopterin and xanthomicrol to have antiangiogenic activity in ex vivo and in vitro models of angiogenesis [7]. Although there is some data suggesting antineoplastic effects for xanthomicrol in mice (data not published), in order to proceed to investigate the in vivo antineoplastic effects of calycopterin and xanthomicrol, one has to have information about pharmacokinetic parameters of these two flavonoids, particularly the maximum attainable plasma concentration after a particular dose and drug half-life. Also any toxic effects they may possess in whole animals will be of great interest. The current work was carried out to furnish such data.

In order to speed up the purification process and increase the productivity of the semi-preparative HPLC purification, the procedure for extraction, isolation and purification of xanthomicrol and calycopterin reported previously [6] was modified. Inclusion of an alumia treatment step after alternate alkali/acid extraction of the flavanoid fraction, helped remove the unmethoxylated hydroxyflavones. This treatment, decreased the complexity of the extract to the extent that a new mobile phase containing triethylamine could be used for the semi-preparative HPLC phase. The solubility of the crude extract in the new mobile phase was about 10 times greater than the previously reported simple mobile phase, which meant that during each semi-preparative HPLC step, 10 times greater quantities of calycopterin and xanthomicrol were obtained. Also, the change in mobile phase helped shorten the run time to 70 min compared to the previously reported 125 min. Taken together, the efficiency of the new purification process was increased by about 20 fold. However, the total recovery of xanthomicrol, calycopterin and circimaritin, were similar to what had been reported previously (Table 1) [6].

During the pharmacokinetic study, one has to withdraw multiple blood samples from the test animal over a few hours. In order to ensure relatively constant physiological homeostasis during the pharmacokinetic study, it is best to withdraw as little a blood sample as possible. This in turn means a small amount of plasma harvested for analytical purposes, typically about 100 μL for the rat, increasing the sensitivity and repeatability constrains of the assay method. Therefore, in order to measure calycopterin and xanthomicrol in rat plasma, a HPLC method was developed and validated that utilized online solid phase extraction, offering enhanced sensitivity and improved low-concentration precision. The method was simple in that plasma deprotination was acheived by addition of zinc sulphate and acetonitrile. The total volume of deproteinated sample was 350 μL, of which, after centrifugation, 250 μL was injected onto the column. In other words, the content of greater that 70 % of the plasma sample was loaded onto the analytical HPLC column, maximizing the total material loaded. Also, the online SPE, concentrated the sample’s flavonoid content, helping tominimize band broadening through the analytical column, again enhancing the detection and quantification limits of the assay (Table 2). Although the quantitation of xanthomicrol or calycopterin in plasma has not been reported before, workers have measured other flavanoids in human or animal serum: Busby et al. [21] used 1000 μL of human serum to measure isoflavones genistein and daidzein. They achieved a LOQ of 1.7 and 3 μg/mL for genistein and daidzein respectively. These values are approximately 30 times higher than those obtained for xanthomicrol and calycopterin in the current work. Biasutto [22] measured quercetin in rat plasma. Using 200 μL samples and a sample deproteination step involving solvent extraction, they achieved a LOQ of 50 ng/mL for this flavonoid [22]. This is similar to the value obtained in the present work, however, their method was more elaborate and time consuming.

The assay thus developed in this study was applied to the pharmacokinetic study of xanthomicrol and calycopterin in rats. The I.V. route for drug administration was chosen because this ensured all administered dose would reach the systemic circulation, without the need to take into account loss during oral absorption and less than complete bioavailability. Therefore the data obtained from I.V. administration of the flavonoids, would give more direct estimates of pharmacokinetic parameters of interest in this study which would enable us to design more valid in vivo antitumor assays for these two agents.

Both agents are relatively hydrophobic and thus do not dissolve in water based solvent systems. A number of solvents which were able to dissolve xanthomicrol and calycopterin were investigated as vehicles for the delivery of these compounds into the blood stream. DMSO was chosen as it is relatively benign in terms of toxicological effects (a number of drug formulations or medical devices containing DMSO are marketed e.g., RIMSO-50 from Bioniche Pharma, USA and Dolicur from Schering, Germany). Also, the volume required as vehicle (200 μL) was small compared to total blood volume in rat (about 20 mL) and would be unlikely to have an appreciable effect on pharmacokinetics of flavonoids being studied.

In order to keep to a predefined sample withdrawal schedule, a cannula was inserted into the rat’s vein at least 24 h before pharmacokinetic study. Cannulation procedure for the femoral artery [14] proved to be easier than that of the carotid artery with faster recovery of the animal after the procedure. Blood sampling schedule was designed to ensure there were enough samples in the initial period after drug administration, during which time changes in drug concentration were likely to be rapid. Plots of the logarithm of both calycopterin and xanthomicrol plasma concentrations verses time were not linear which suggested that the one compartment model would be unsuitable for analysis of concentration data. Two compartment analyses were successfully applied.

Calycopterin has one methoxy group more than xanthomicrol and is thus expected to be more hydrophobic. This is confirmed by the longer retention time of the former during reverse phase chromatography (Fig. 3). However, the extent of the effect of this increased hydrophobicity on disposition of calycopterin in rat, was unexpected. When administered at 20 mg/kg, xanthomicrol had a calculated initial plasma concentration of 2345 μg/L while the corresponding value for calycopterin was 312 μg/L (Table 3). This is probably because, compared to xanthomicrol, calycopterin distributes more rapidly into various organs thus leaving blood or the “central compartment” in our model, more quickly. This, which is also reflected in the difference in the V_c_ values for these flavonoids (Table 3), is also confirmed by the difference in the K_12_ rate constants. The rate constant for transfer of calycopterin into the central compartment is smaller than that of xanthomicrol which suggests a tendency for calycopterin to distribute more easily into tissues. The elimination rate constants are not very different, which may explain similar t_1/2_ of elimination for these two flavonoids (Table 3).

**Fig. 7:**
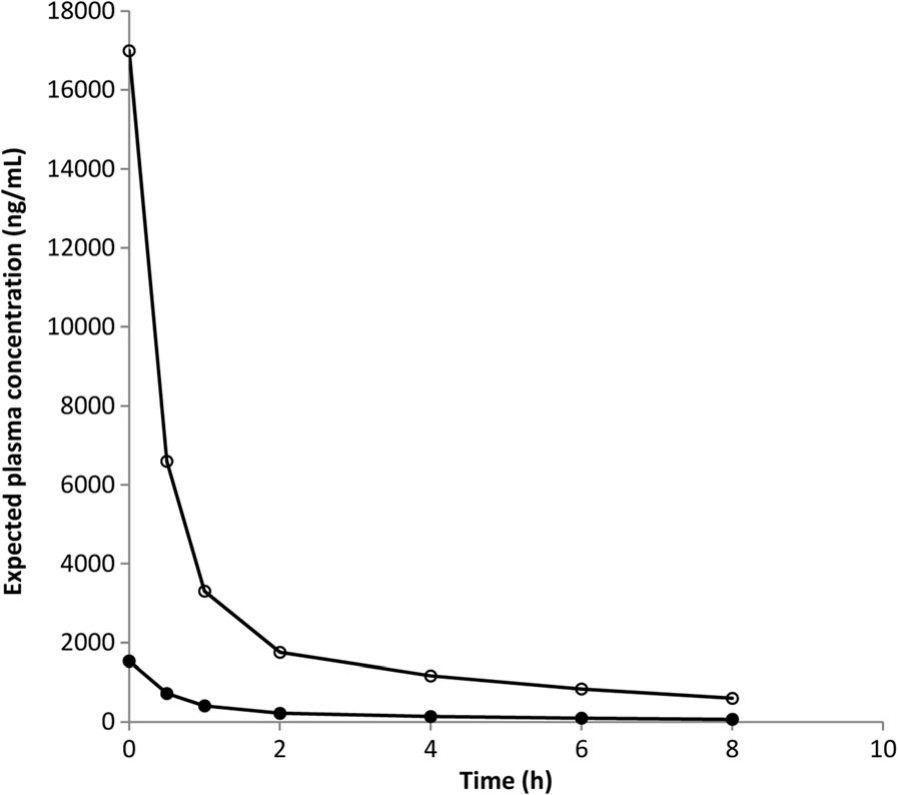
Predicted plasma calycopterin (●) and xanthomicrol (○) concentration-time profiles after a single IV bolus dose of 30 mg/kg or either compound in the rat. The values were calculated based upon the two compartment models developed in this work

Our previous research had shown that at 500 ng/mL, both calycopterin and xanthomicrol inhibited capillary-like tube formation by HUVEC cell [7]. Our aim in this work was to find doses of these flavonoids which could produce such plasma levels over extended periods. This would let us investigate the in vivo effects of these compounds on experimentally induced tumors at therapeutically relevant plasma concentrations. The models generated, were used to predict the plasma concentration-time profiles for different doses of calycopterin and xanthomicrol (Fig. 7). After a single I.V. dose of 30 mg/kg, xanthomicrol plasma concentration would be greater than 500 ng/mL for more than 6 h while calycopterin’s plasma concentration would dip below 500 ng/mL 30 min after administration.

**Fig. 8:**
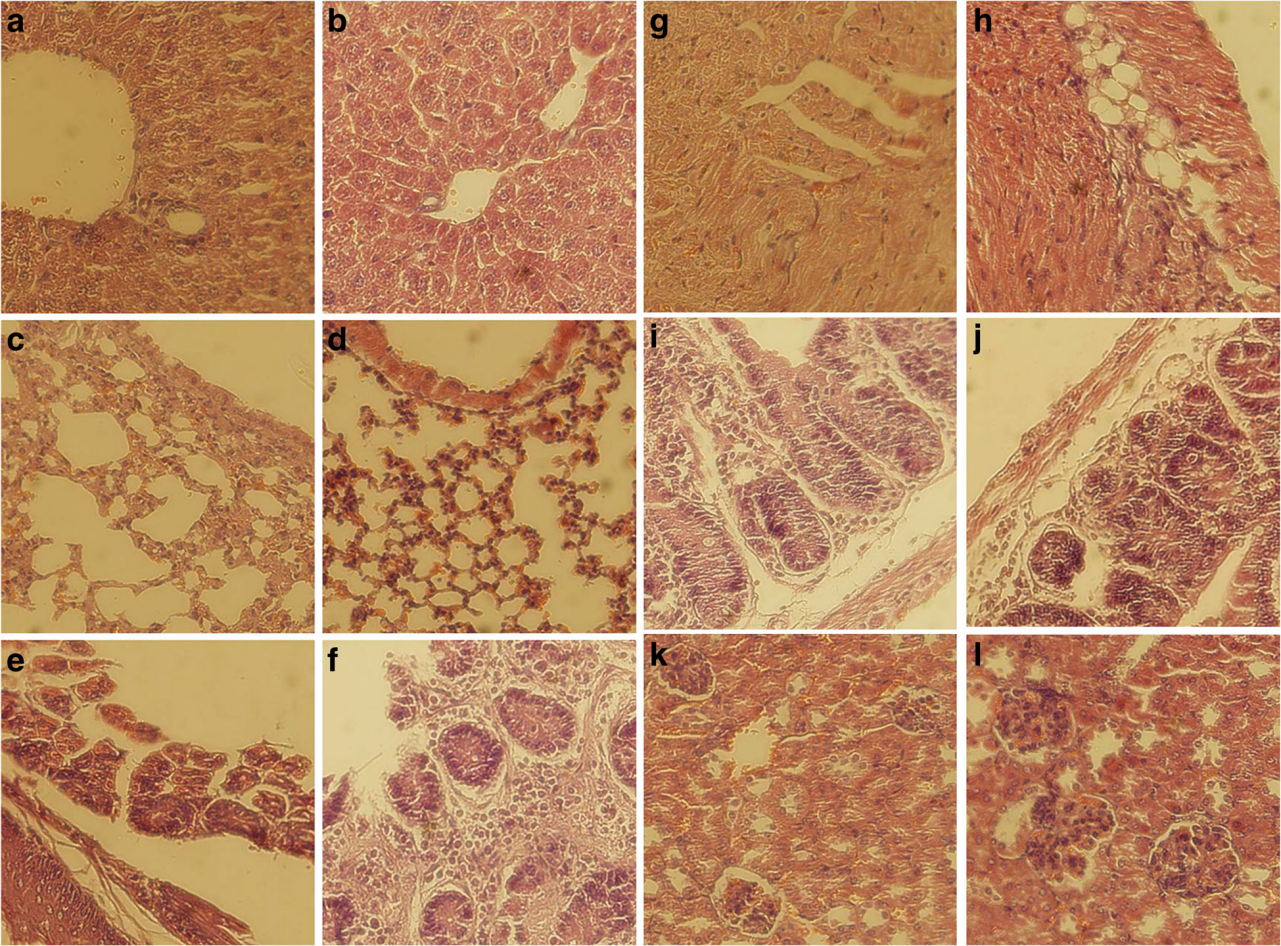
Effect of xanthomicrol treatment on vital organ histology in mice. Images on the left have been selected from animals treated with 50 mg/kg xanthomicrol and images on the right are from control animals. **a** and **b** Liver, **c** and **d** Lung, **e** and **f** Stomach wall, **g** and **h** Heart muscle, **i** and **j** Small intestine, **k** and **l** Kidney

The pharmacokinetic superiority, in terms of maximum attainable concentration and the time it took to leave the central compartment, meant that xanthomicrol had the greater potential as an anti-angiogenic agent. Thus, the potential toxic effects of xanthomicrol at 30, 40 and 50 mg/kg/day for six days, were studied. As none of the animals displayed xanthomicrol induced toxic effects with in the first 24 h after the highest dose, this flavonoid appears not to have acute toxic effects. None of the animal died in any of the administered dose groups. After six days of drug treatment, none of the animals AST levels, which are indicative of liver damage, were not significantly different amongst control, vehicle and test groups. Histological examination (Fig. 8) was unable to find xenobiotic induced damage to vital organs. There was no change in animal weights compared to control group and no animal showed any signs of motor or behavioral abnormalities. Xanthomicrol, at administered doses, did not appear to have toxic effects in mice. However, it is important to remember that this study did not attempt to determine LD_50_ value for xanthomicrol or show long term safety. Future toxicological studies must determine LD_50_ and also examine sub-acute and chronic toxic effects.

## Conclusion

Both calycopterin and xanthomicrol have anti-angiogenic properties however, our data suggests that from a pharmacokinetic point of view, xanthomicrol has greater potential as an antitumor agent.

## Abbreviations

AST: Aspartate transaminase
ALT: Alanine transaminase
DMSO: Dimethyl sulfoxide
SPE: Solid phase extraction
VEGF: Vascular endothelial growth factor
